# Alzheimer’s disease classification using a hybrid deep learning approach with multi-layer U-net segmentation and XAI driven analysis

**DOI:** 10.1371/journal.pone.0332572

**Published:** 2025-09-29

**Authors:** Muhammad Zubair, Arfan Jaffar, Sadaf Hussain, Sheeraz Akram

**Affiliations:** 1 Faculty of Computer Science and Information Technology, The Superior University, Lahore 54000, Pakistan; 2 Faculty of Computer Science, Lahore Garrison University, Lahore 54000, Pakistan; 3 Information Systems Department, College of Computer and Information Sciences, Imam Mohammad Ibn Saud Islamic University (IMSIU), Riyadh 11432, Saudi Arabia; UO: University of Okara, PAKISTAN

## Abstract

Alzheimer’s disease (AD) is a neurodegenerative illness causing a significant decrease in cognitive function, and early, accurate diagnosis is of great therapeutic and diagnostic value. Currently, there is promising potential for applying various types of artificial intelligence techniques, such as enhanced models of deep learning, for classifying Alzheimer’s disease. Therefore, this study proposes an Outline of deep learning to classify Alzheimer’s disease with segmentation using the Multi-Layer U-Net and a hybrid classification approach combining multi-scale EfficientNet with SVM. The proposed methodology consists of a four-phase process: (1) Whole brain segmentation, (2) Gray matter segmentation using multi-layer U-Net segmentation, (3) Feature extraction using Multi-Scale Efficient Net with SVM for classification, and (4) XAI (explainable AI) techniques by integrating Saliency Map Quantitative Analysis for increased clinical trustworthiness and model interpretability. It is found that the experiment results provide promising classification performance for three classes – Alzheimer’s Disease (AD), Mild Cognitive Impairment (MCI) and Cognitively Normal (CN) with an overall accuracy of 97.78% ± 0.54%, precision of 97.18% ± 1.14% (AD), 97.78% ± 0.29% (CN) and 97.03% ± 1.10% (MCI), recall of 97.90% ± 0.77% (AD), 97.49% ± 1.34% (CN) and 97.25% ± 0.99% (MCI), and F1 score of 97.74% ± 0.63% (AD), 97.78% ± 0.79% (CN), and 97.54% ± 0.69%(MCI). The results obtained underscore the elegance of the proposed approach in correctly classifying Alzheimer’s disease stages. Future work will evaluate the model on publicly accessible Alzheimer’s disease MRI datasets and incorporate advanced XAI techniques for increased interpretability and diagnostic reliability. The work focuses on Human Health.

## Introduction

Alzheimer’s disease (AD) is a neurological disease that causes dementia, loss of memory, and impaired decision-making. It is the most prevalent form of dementia on the globe and is most prevalent in older adults [1]. Those pathological processes include an accumulation of amyloid beta (A) plaques, the development of tangled tau proteins, and the loss of neurons, which leads to cortical and functional synaptic failure [2]. Though the minimum age of occurrence of AD is 65 years, its early onset becomes evident at an earlier period of life. As a rule, the development of the symptoms is gradual, and the symptoms can affect memory, reasoning, and executive functions. The differentiation of AD with similar dementias that include vascular dementia, Lewy body dementia, and frontotemporal dementia is based on progressive and systemic dissemination: the pathology originates in the hippocampal system. It moves into changes in the cortical and subcortical areas after the disease has spread extensively, with neuronal malfunction and widespread brain atrophy. Due to this gradual retrogression, it may not only be hard to diagnose but also to manage. AD has severe medical, social, and economic repercussions in public health terms. The World Health Organization (WHO) estimates that the cases of dementia will increase almost threefold, reaching an estimated 139 million in 2050 compared to about 55 million in 2023, and there is an expected sharp increase in middle-income countries [3]. AD causes almost 70% of all dementia cases, and its current economic cost is almost 1.3 trillion dollars, which will increase to 2.8 trillion dollars by 2050. With these statistics, AD is a developed public health issue and a rapidly expanding research objective [4]. The impact of Alzheimer’s disease (AD) extends well beyond healthcare and regards issues such as being able to live personally, as well as finding employment. Average family members and paid caregivers devote about 22 hours a week helping AD patients, which amounts to emotional stress, financial concerns, and disruption of careers [5]. The overall expenditure on AD in the U.S is more than 355 billion dollars, where all the long-term care is combined with the costs in hospitals and out-of-pocket expenses. The studies also reveal that patients with AD are hospitalized approximately twice as compared as those without dementia. All these studies have not enabled scientists to determine that the origin of AD is a single factor. The picture of the risk is complicated: genes are important, the environment contributes, and lifestyle choices do matter as well. Particularly important are the factors of genetics. Individuals who have been affected by APOE 4 or have alterations in APP, PSEN1, or PSEN2 are susceptible to a greater risk of AD [6]. Collectively, these genetic risk factors are estimated to contribute about 115% of AD, and most of them induce symptoms 65 years before, a type of the disease referred to as familial Alzheimer’s disease (FAD) [7]. Also, disease progression is contributed to by environmental and psychological factors. Elevated cortisol has been associated with chronic stress, and it has been linked to increased cortisol that accelerates hippocampal atrophy and can accelerate cognitive decline [8]. Medication and lifestyle choices that could also have a great influence on AD risk are also emerging evidence. Long-term drug use of benzodiazepines, antihistamines, and opioids has been reported to cause cognitive impairment and an increased predisposition to develop Alzheimer’s [9,10]. In addition, modifiable lifestyle factors, including physical inactivity, poor nutrition, and smoking, contribute to the rapid propagation of vascular damage and neurodegeneration as well as neurocognitive decline [11]. Therefore, these findings underscore the importance of multi-disciplinary AD prevention that links genetic investigation to lifestyle interventions and early diagnostic strategy to avoid disease progression. Alzheimer’s disease has three major stages, each of which the disease affects the cognitive and motor function. The Cognitively Normal (CN) stage consists of those with mild signs of cognitive impairment but without obvious clinical signs; small brain changes may begin years before occurrences of symptoms. In Mild Cognitive Impairment (MCI), there is subtle memory loss, difficulty with words, and possibly mild confusion; the hippocampus and entorhinal cortex, which help form memory, are affected. As the disease progresses to the AD stage, people will experience severely changed personalities, high levels of confusion and disorientation, and will become very dependent on others. In this stage, brain shrinkage is widespread, and cortical atrophy severely affects speech, mobility, and overall cognitive function. These stages are essential for early diagnosis, early intervention, and designing a good care plan. There are also distinct brain imaging changes as observed with structural degeneration of both gray matter and white matter in Alzheimer’s disease. The most characteristic feature of AD is gray matter (GM) degeneration involving the hippocampus, prefrontal cortex, and entorhinal cortex, which are necessary for memory processing and cognitive functions [12]. In parallel, white matter (WM) disruptions, loss of myelinated axons affecting neural connectivity, produce delayed information processing and confusion, cognitive decline [13]. Cerebrospinal fluid (CSF) biomarkers, e.g., altered levels of β-amyloid, tau, and phosphorylated tau proteins, are very important as measures of early Alzheimer’s disease diagnosis [14]. Inter-part mental/inter-disciplinary collaborations are becoming more frequent in using recent advancements in machine learning, with physics-informed neural networks (PINNs), to reconstruct biomedical phenomena that are defined and governed by underlying physical laws-- whether these be the fluid dynamics of blood flow or the volumetric expansion of a tumor. We are directly interested in structural MRI and deep-learning methods that allow automatic segmentation and classification of various structures; however, the potential use of PINNs also seems interesting to address by examining the spatiotemporal evolution of neurodegenerative disorders progression. Various neuroimaging procedures are unavoidable tools for identifying and tracking the progress of Alzheimer’s disease (AD). Each of those modalities provides different information about the progression of a disease. Magnetic Resonance Imaging (MRI) provides high resolution, structural information, and thus diagnosis is easy in terms of gray-matter atrophy and hippocampal shrinking- important biomarkers in AD. Convolutional Neural Networks (CNNs), which take the form of deep-learning models, have provided approximately 95% of the accuracies in classification [15]. The positron emission tomography (PET), on the other hand, can visualize the accumulation of β-amyloid plaques as well as their tau protein aggregates. These pathological characteristics have been detected by using recent deep-learning-based methods that automatically reveal these characteristics in PET scans, thus increasing the chances of early diagnosis [16]. Recurrent neural networks (RNNs) and convolutional models have been explored to assess brain activity by applying electroencephalography (EEG) to detect abnormal patterns of neurons in AD [17]. Whereas Computed Tomography (CT) is largely used in later stages of the disease to identify pronounced brain atrophy, research proves that the use of deep learning can be beneficial in perceiving structural degeneration in CT-based images as well. As a result, the adaptation of AI methods to a wide range of imaging is not only more powerful but also a broader system that allows for detecting AD early and correctly. Recent developments in the field of deep learning insinuate that, in the not-so-distant past, it will be possible to query MRI scans to arrive at the differentiation of Alzheimer’s disease. CNNs are widely used in Alzheimer’s disease diagnosis due to their ability to extract spatial features from structural MRI images. Both 2D and 3D CNN architectures have successfully classified subjects into AD, MCI, and CN [18]. Vision Transformers (ViTs) have also gained attention for their ability to capture global contextual information in MRI scans. While CNNs are effective at identifying local features, ViTs complement them by modeling long-range dependencies. All these advances provide a viable framework in which hybrid stroke segmentation techniques like the one here can merge the high sensitivity of brain regions segmentation with good, strong feature extraction and classification techniques.

The Main Objectives of this study are as follows:

- Develop a hybrid deep learning model combining Multi-Layer U-Net and Multi-Scale EfficientNet for Alzheimer’s diagnosis from MRI Scans.
- Segment the gray matter brain region using a Multi-Layer U-Net to focus on structural changes relevant to AD.
- Improve classification accuracy using Multi-Scale EfficientNet on segmented MRI regions.
- Apply explainable AI (XAI) to enhance clinical interpretability.

In this study, we address the challenge of accurate Alzheimer’s Disease Classification by integrating segmentation, feature extraction, and model interpretability into a unified framework. Motivated by the need for precise analysis of brain regions and explainable results, we propose a hybrid deep learning approach that combines a multi-layer U-Net for gray matter segmentation, Multi-Scale EfficientNet for feature extraction, and SVM with grid search for classification. Furthermore, to enhance clinical trust, we apply Explainable AI(XAI) techniques that visually interpret model predictions. This integrated pipeline improves diagnostic accuracy while addressing the common limitation of traditional black box approaches.

## Literature review

Timely and precise diagnosis of Alzheimer’s disease (AD) is crucial since AD is a major cause of dementia globally. The improvement of AD detection and classification has been demonstrated through various computational techniques, mainly with the help of deep learning. This review thus concentrates on DCNN-based AD diagnosis applications by reviewing recent applications of DCNNs for image segmentation, classification, and explainable AI (XAI) techniques. In this work, we also discuss how these DCNN-based methods are being used to explore patterns in brain MRI scans that can be correlated with AD and deliver more interpretable, trustworthy diagnostic tools to clinicians. Mohammed et al. [19] proposed a deep-hybrid learning model that combines AlexNet, ResNet-50, feature unification, and an SVM model to detect Alzheimer’s disease (AD) with an accuracy of 94.8%. The proposed methodology, based on combining deep and traditional machine-learning approaches, was compared to other common methods, stating that it was more accurate and efficient than other methods to be use in diagnostics. However, the model was limited in that it needed pre-designed networks to work, which did not have the capability of extracting multiscale features and did not openly discuss issues of domain generalization across heterogeneous MRI datasets. Addressing these issues, except for these limitations, is the key goal of the current framework. Sharma et al. [20] propose a mixed model that combines transfer learning and an ensemble voting process to classify magnetic resonance imaging (MRI) data as Alzheimer’s disease. Their approach achieves an accuracy of 91.75%, 96.5% specificity, and an F1-score of 90.25%. The use of DenseNet as a feature extractor shows positive effects on the accuracy of predictions; the use of ensemble voting, though, undermines interpretability and generates additional computational expense at the inference stage. In addition, the authors do not assess the correspondence of the model on the datasets collected under another protocol, which is essential in clinical deployment when there is a need to have an effective generalization of data collected using diverse protocols. The automated pipeline of Alzheimer’s identification in magnetic resonance imaging (MRI) was suggested by Ghazal et al. [21] based on transfer learning. They used AlexNet with a multi-class classifier that could distinguish different disease stages by training the model on images and attained 91.70% accuracy based on this classification. The method stands out since it tackles the multi-class classification as well as specifically tailors a deep net to an issue. Still, the work does not offer an in-depth study of spatial feature hierarchies and the study does not contain any cross-dataset generalization The study by Pradhan et al. [22] that followed reached the precision of classification at 99.05% using deep learning and a combination of multimodal information, namely, electronic health records and single-nucleotide polymorphisms (SNPs), thus demonstrating the potential application of heterogeneous information sources to increase the performance. It is, however, computationally expensive and cannot be used unless a large amount of data is available, and the research is therefore not very practical to scale.

The study of S. M. Mahim et al. [23] advances the existing research on the diagnosis of Alzheimer’s in a manner that proposes a novel approach that combines Vision Transformer (ViT) and Gated Recurrent Unit (GRU), considerably enhancing the level of diagnostic precision and the interpretability of such a method. Although the model proves to be quite promising, its mathematical complexity and high training burden make it inappropriate to be used in clinics in real time.

Mahmud et al. [24] present a transfer ensemble framework, ultimately attaining an accuracy of 96% and focusing on promoting transparency using saliency maps and Grad-CAMs. However, little work on generalization across different sets of data can be done by the study. Suchitra et al. [25] use EfficientNetB7 as a general image in transfer learning on the ADNI dataset and achieve 98.2% accuracy. The approach does not, however, provide a comparative assessment of the conventional classifiers, nor does it integrate the benefits of feature representation at multiple scales. Comparatively, though, both multi-scale EfficientNet and Support Vector Machine (SVM) are incorporated in the present form of study, thereby improving both accuracy and generalization together while remaining efficient on the computational front.

El-Assy et al. [26] proposed a novel architecture of CNN using the ADNI dataset based on MRI Images for multi-class AD classification, achieving accuracy of 99%, 99.5%, and 99.13% for three, four, and five category classification. The model combines CNNs with pooling layers and distinct filter sizes, demonstrating high efficiency in early and precise classification of AD stages. Liu et al. [27] proposed a CNN-based framework with 3D DenseNet for hippocampal segmentation and AD classification, achieving 87% Dice similarity and 88.9% accuracy for AD vs NC classification using the ADNI dataset. Balasundaram et al. [28] utilized hippocampus segmentation to compare models trained on segmented regions versus full pictures. Using Kaggle and the OASIS 2 dataset, which applied deep learning and ensemble learning, to achieve early diagnosis with classification accuracy as the primary metric. Battula et al. [29] proposed an Alzheimer’s disease diagnosis method using Deep ResUnet for segmentation, MASNet for feature extraction, and EfficientNetB7 for classification. Tested on the Kaggle and ADNI datasets, the methods achieved accuracies of 99.31% and 99.38%. Steinbart et al. [30] developed and validated manual and automatic piriform cortex segmentation methods using MAPER with Alzheimer’s disease (AD) and temporal lobe (TLE) for patients. Emmanuel et al. [31] suggested a hybrid model, SegResNet, combining SegNet for segmentation and ResNet-101, using structural MRI data for classification to detect Alzheimer’s disease (AD. The model outperformed existing methods like KNN and ACS, achieving superior classification accuracy by leveraging segmented MRI images as features for AD, Cognitive Normal (CN), and Mild Cognitive Impairment (MCI) categorization. Biswas et al. [32] proposed a system for Alzheimer’s disease detection using 3D MRI, classifying severity as Normal, Mild, and Severe. The model achieved 92% accuracy on the ADNI dataset and 99% on the OASIS dataset with Random Forest. Sait et al. [33] proposed an Alzheimer’s detection model combining LeViT, EfficientNetB7, and XGBoost. Achieving 99.8% accuracy on the OASIS dataset, outperforming existing methods, and aiding early AD detection. Mahmud et al. [24] proposed an explainable AI approach for Alzheimer’s disease diagnosis using deep ensemble learning and transfer learning approaches, achieving 96% accuracy. The model incorporates XAI procedures to enhance interpretability, providing clinicians with valuable insights into decision-making processes. Naseer et al. [34] developed an improved U-Net-based form for lung cancer classification, utilizing portion segmentation and classification with a modified AlexNet-SVM. The model achieved a high performance with 97.98% accuracy and 97.70% F1 score on the LUNA dataset. Bawil et al. [35] A specialized gray matter segmentation method was developed for classifying white matter hyperintensities (WMH), including juxtacortical WMH (JCWMH), based on a conditional generative adversarial network (cGAN) and reached a Dice similarity coefficient (Dice) of 0.76. The major reduction in T1 dependence provided by this study substantially decreases dependence on T1 images while still classifying WMH via FLAIR images alone, thus streamlining the neurodegenerative disease research. Kumar et al. [36] present an average dice ratio of 89.74% on the IBSR dataset as the model and innovatively introduced skip connections, making use of fine-grained multiscale information to help in the identification of the tissue boundary. Lvovsky et al. [37] proposed an adaptive U-Net model for the automatic Extraction of white matter, gray matter, and cerebrospinal fluid from brain MRI scans. To improve the deep learning applications for medical imaging, the method was shown to have superior performance than existing approaches by exploiting a mixture of kernel sizes in feature extraction and a Local Convolutional Neural Network for the classification of uncertain regions. Shibata et al. [38] developed a 3D residual UNET convolutional neural network model for the automatic segmentation of the hippocampus and calculation of magnetic susceptibility in Alzheimer’s disease. The authors show that hippocampal volume correlates strongly with magnetic susceptibility, providing a bolster for the assessment of pathological changes of neurological disorders. It shows a possibility of deep learning for enhanced imaging studies. Kumar et al. [39] proposed an innovative framework for early Alzheimer’s Disease diagnosis using Adaptive Moving Organizing Map (AMSOM) integrated with K-means clustering and PCA for better tissue segmentation and classification. The method was impressive at the accuracy level of 99.8%, improving over the current techniques. Using dynamic neuron weight adjustments to refine image segmentation in this study adds the tactic to the growing list of joinable AI that can be used for robust diagnostic applications. Alex et al. [40] utilized a hybrid learning model of 3D convolutional neural networks fed with cerebrospinal fluid biomarkers for the later (further) early detection of Alzheimer’s disease. Finally, they were able to demonstrate how much this had improved their ability to identify people with mild cognitive impairment rather than Alzheimer’s disease, as a promising method to pick up the disease early. Asaduzzaman et al. [41] proposed the ALZENET model, an ensemble deep learning model that predicts Alzheimer’s disease from MRI analysis and presents an impressive accuracy of 97.31% on the test. In the case of an imbalance of classes, the study then integrates synthetic data generation to solve a common challenge in Alzheimer’s research. Najjar et al. [42] established a hybrid deep learning model, based on U-Net and VGG16, for extracting hippocampus localization in Alzheimer’s diagnosis. Empirically, they showed that data augmentation and transfer learning techniques can improve the accuracy in identifying affected brain regions compared to other methods of diagnostic methods. Akan et al. [43] proposed ‘ViTranZheimer’, where the video vision transformers are used to examine 3D brain MRI data for the diagnosis of Alzheimer’s disease. This provides an accuracy of 98.6% higher than traditional CNN-BiLSTM and ViT-BiLSTM models. It importantly captures inter-slice dependencies effectively (frameworks that treat MRI slices as frames in a video give a better diagnosis precision). Hagos et al. [42] proposed A different multi-modal deep learning approach combining MRI and FDG PET scans for Alzheimer’s disease identification, with a reduction in the use of FDG PET by up to 92% with no loss of accuracy. The model accounts for uncertainty estimates to mimic how physicians would make a clinical decision: only signing for an FDG PET scan when the uncertainty is low [43,44]. Integration of biomedical and computational strategies has shown a measurable impact in enhancing Alzheimer’s disease research. Drug repurposing techniques demonstrate potential to accelerate therapeutic discovery, while cloud-based machine learning models offer scalable and efficient diagnostic workflows. To consolidate the findings from the reviewed studies and enable easier comparison, Table 1 presents a summary of selected works highlighting key metrics such as Accuracy, Specificity, XAI Used, dataset, Model, and identified limitations.

**Table 1:** Summary of recent Alzheimer’s classification studies.

Publication	Model Type	Dataset	Accuracy	Specificity	Interpretability	XAI	Limitations
Mohammed et al. [19]	AlexNet + ResNet 50 Svm	Oasis	97.2	93	No	No	Manual diagnosis relies on expert-defined features
Sharma et al. [20]	DenseNet+ Voting Classifier	MRI	91.75	96.5	No	No	Focus only on feature-level vision
Ghazal et al. [21]	Transfer learning (AlexNet)	MRI	91%	Not Mentioned	No	No	Lack of Multi-class Performance
Pradhan et al. [22]	Multi-modal deep learning	MRI	99%	Not reported	No	No	High complexity, difficult to generalize
Mahmud et al. [24]	Ensembel+Xai	ADNI	96	Not Mentioned	Yes	Grad-Cam	Computationally intensive ensemble interpretability depends on visual analysis.
Suchitra et al. [25]	Transfer Learning	ADNI	96%	Not Mentioned	NO	NO	No interpretability Support
El-Assy et al. [26]	Custom CNN	ADNI	99%	Not Mentioned	No	No	Not tested across diverse datasets
Liu et al. [27]	CNN + 3D DenseNet	ADNI	88.9%	Not mentioned	No	No	Requires accurate hippocampal segmentation
Balasundaram et al. [28]	Segmentation+ CNN+Ensembele	OASIS, Kaggle	98	Note mentioned	NO	NO	Limited due to dataset diversity
Biswas et al. [32]	ML + 3D MRI Classification	ADNI	99%	No	No	No	May not scale well on a large 3D dataset
Kumar et al. [45]	U-Segnet	ISR	89%	Not mentioned	No	No	Limited training data

The systematic review of the existing literature on machine learning in the field of hippocampal segmentation suggests that, according to the general trend of results, the study revealed several recurring weak aspects. Among these limitations are; (i) dependency on accurate segmentation of the hippocampus to provide accuracy of the model [24], (ii) the lack of external validity of the method to distinct datasets [27], (iii) a need to carry out manual segmentation with ability of experts to validate the segmentation finding [29], (iv) sensitivity of the method to the initial quality of a segmentation, and, (v) a practical challenge to apply the methodology on large-scale 3D MRI studies [31]. When combined, these results indicate that there will always be weaknesses relating to the accuracy of segmentation, the robustness of the models, and scalability. The solution to these problems requires the construction of a framework, which at the same time is robust, explainable, and capable of generalizing across datasets, an effort that is described in the research presented next in detail.

Based on analysis of the available literature, recent deep-learning strategies have shown a significant degree of accuracy by being applied to the detection of Alzheimer’s disease using MRI data, specifically using CNN model-based architecture, the architecture of hybrid models, as well as transfer-learning supported approaches. Convolutional neural networks like AlexNet, DenseNet, and EfficientNet have been proven to have strong feature-extraction properties and have been used widely in both binary classification and multi-classification jobs. However, most CNN-based approaches are quite susceptible to large-scale, annotated datasets and exhibit poor explainability, which is of great clinical significance. Hybrid models (i.e., using segmentation networks, e.g., SegNet, U-Net, combined with classifiers, e.g., ResNet, EfficientNet, SVM) enhance segmentation accuracy as the models attend to relevant areas of the disease and tend to produce impressive results, but result in structural complexity and lengthy training times. People have turned to transfer learning because it would shorten training costs and make use of the learned weights, whereas the mismatch between the features of natural images and medical imaging may limit their versatility. At the same time, although the latest studies have employed interpretability mechanisms, including Grad-CAM and saliency maps, most deep-learning solutions still act as a metaphorical black box, and thus create a problem of clinical trust. All these shortcomings highlight the need to have an effective, comprehensible, and balanced structure, which our proposed model aims to solve. While recent studies have integrated Grad Cam and other XAI techniques for improved interpretability, many models still lack clinically aligned or region-specific explanations and operate as black boxes. Although B. Bohle et al. [46] have explored pixel-wise explanation in Alzheimer’s disease (AD) classification, such techniques often suffer from poor spatial resolution, inconsistent visualizations, and limited clinical interpretability. These limitations, particularly in segmentation precision and clinical transparency, point to key gaps in current deep learning methods, which our proposed hybrid framework aims to address.

Furthermore, A constant research problem is how to use fine-grained segmentation of anatomical structures. Most state-of-the-art methods skip the classification step or use basic, one-layer architectures that are likely to miss the differences in gray matter. At the same time, explainability is underutilized or at best considered a peripheral issue, thus weakening the clinical credibility of otherwise highly performing systems. To counter these inadequacies, the proposed study posits a new, interpretable, and balanced architecture of a convolutional neural network that considers both the aspect of computational complexity as well as the transparency of the model.

Building on these insights, we present our method, which integrates segmentation, classification, and interpretability in a unified pipeline.

The key contributions of this study are outlined as follows

Automated and Explainable AD Classification: In this research, a novel framework of automated and explainable AI Analysis based on multi-layer U-Net segmentation, Multi-scale EfficientNet classification, and saliency map visualization is proposed.

**Fig 1:**
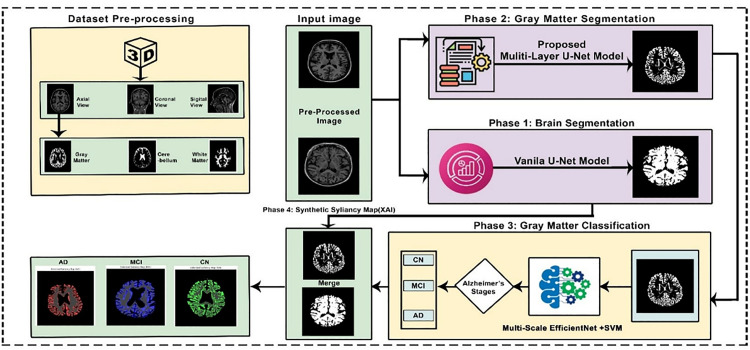
Proposed hybrid framework for Alzheimer’s disease classification.

The overall workflow of the proposed framework is illustrated as shown in Fig 1. Develop a multilayer U-Net architecture for accurate brain segmentation and gray matter segmentation, which makes better use of details within regions as well as of variations within the neighboring regions. Compared to previous methods. A Multi-Scale EfficientNet with an SVM Classifier is used to achieve robust classification based on the merits of various deep learning models to enhance accuracy and generalizability. Integrate Explainable AI (XAI) Techniques, namely saliency maps, to enable the visualization of the model’s decision-making process and increase transparency as well as clinical trust. The goal of this research is to solve the limitations of the previous research and achieve a comprehensive and interpretable approach to AD classification to facilitate a more accurate and confident diagnostic tool.

### Data-preprocessing

**Fig 2:**
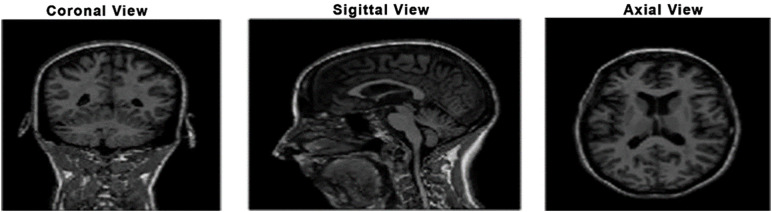
Original 2D MRI views of the brain.

**Fig 3:**
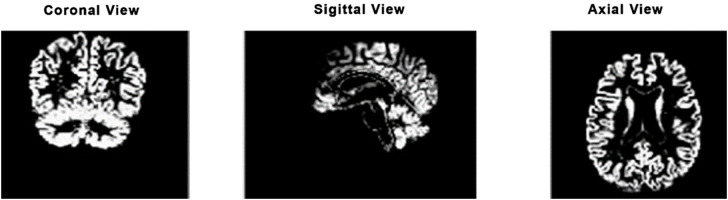
Gray matter segmented views of the brain.

During the data preprocessing stage of this study, we generated brain masks using thresholding techniques, which facilitated the preparation of the dataset for further analysis. This method aligns with the findings of Smith et al. [42] , demonstrating the effectiveness of adaptive thresholding in brain image segmentation. By isolating the region of interest through thresholding, we enhanced the accuracy of our neuroimaging assessments and improved the overall visualization of brain structure. Numerous White matter (WM), Gray matter (GM), and Cerebrospinal (CSF) segmentation [47–49] techniques have been proposed and generally categorized into mathematical and machine learning based tools such as Statistical Parametric Mapping (SPM) and FMRIB Software Library (FSL) are frequently used within the mathematical group [50,51]. Specifically, SPM employs Gaussian models to smooth intensity variations and performs nonlinear registration in conjunction with TPMs to achieve good segmentation. The data set used in this study was obtained from the Alzheimer’s Disease Neuroimaging Initiative (ADNI) database. A total of 306 3D T1-weighted MRIs were used in this study, for which the scans were classified as Alzheimer’s Disease (AD), Mild Cognitive Impairment (MCI), and Cognitively Normal (CN) [47] defined in Table 2. The dataset contains male and female subjects aged 50 to 90 years old. To ensure consistency, all the MRI images underwent skull stripping to remove non-brain tissues for further processing. Due to the significance of the axial plane in Alzheimer’s diagnosis, axial slices were extracted from each 3D MRI volume so that brain structures are represented same way across subjects. 40% of middle slices containing the most important brain anatomy were retrained, excluding peripheral slices with limited gray matter representation. All the preprocessing pipeline steps were completed by the Statistical Parametric Mapping (SPM12) toolbox, a commonly used tool for brain imaging analysis. The co-registration step in this process aligned the MRI scans to a standard template, so all the subjects were aligned in space. It was followed by segmentation, where the brain was divided into Gray Matter (GM), White Matter (WM), and Cerebrospinal Fluid (CSF) based on Tissue Probability Maps (TPM) from the ICBM brain template. After segmentation, the focus was on extracting grey matter (GM) images for further analysis because GM is the most clinically relevant region in Alzheimer’s disease, as it is the primary region in which neurodegeneration takes place. Therefore, GM weighting should be emphasized in Alzheimer’s classification, especially for the early stage of the disease [38]. Each slice was resized to 224 x 224 pixels to meet the input requirements of the network. Normalization was applied on a per-slice basis to ensure consistent brightness and contrast across all scans. The original 2D extracted views, axial, sagittal, and coronal, are illustrated as in Fig 2, and the corresponding gray matter views of each anatomical plane are shown in Fig 3

**Table 2:** Original dataset composition (Raw from ADNI).

Class	Number of subjects
AD	58
CN	115
MCI	133
Total	306

The original dataset comprised 306 MRI scans, and the distribution of images across classes is summarized in Table 2.

After segmentation with SPM12, GM mask images were extracted from axial slices, producing the following image distribution, which is summarized in Table 3

**Table 3:** GM Mask image count after preprocessing.

Class	Number of subjects
AD	289
CN	280
MCI	281
Total	860

For classification purposes, data augmentation was applied using the Keras Image Data Generator. Augmentation techniques include small rotations (±7°) and zoom (±10%) to address class imbalance. After augmentation, each class was expanded to 1600 gray matter mask images used for classification purposes. The augmented dataset was then split into 60% training, 20% validation, and 20% test set. The ratio was chosen for balanced evaluation while retaining enough data for training.

### Proposed methodology

Alzheimer’s disease (AD) reduces memory and other cognitive functions in a long and relentless way. Early diagnosis is necessary for prompt intervention and suitable therapy. Magnetic Resonance imaging (MRI) has been demonstrated to be a powerful imaging method in the diagnosis and the monitoring of the progression of Alzheimer’s disease. Pathological changes associated with AD and brain structure segmentation. This paper introduces an advanced framework for Alzheimer’s disease detection based on deep learning models to perform brain and gray matter segmentation, followed by classification applied from an advanced hybrid model. Furthermore, Explainable AI techniques are used to increase the model’s interpretability of its predictions. The proposed methodology is divided into four distinct phases: **(1) Brain segmentation**, where the Vanila U-Net model is utilized to segment brain region from MRI scan; **(2) Gray matter segmentation**, where multi-layer U-Net model is applied to extract Gary matter region; **(3) Classification,** which involves the use of the advance hybrid model for categorizing the segmented images and **(4) Explainable AI,** where the Modified synthetic saliency Map is applied to visualize and interpret the model decision-making process, Each Phase is described in detail in the following section, supported by visual block diagram (Fig 1) providing a comprehensive framework for Alzheimer’s disease detection and analysis.

#### Phase 1: Brain segmentation.

**Fig 4:**
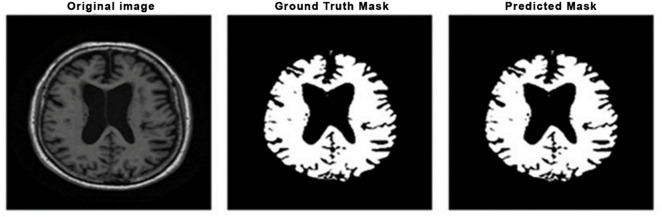
Visual comparison of Brain segmentation results.

In the first stage of segmentation, a standard U-Net without enhancement (Vanilla U-Net) model is used for segmenting the brain. The Vanilla U-Net model has been deployed due to its strong boundary detection capabilities in medical imaging tasks for the purpose of segmentation. The use of its encoder-decoder structure with skip connections and the ability to retain the spatial information makes it highly fitting for segmenting complex brain structures from MRI scans. Furthermore, learning capabilities have been used in this study, which benefits effective learning from limited datasets without requiring extensive pretraining. The model was fed the original MRI and was told to output a binary mask of the segmented brain region in the training process. Fine-tuning or modification of the standard U-Net architecture was not employed. As seen in Table 4, the model achieved 99% accuracy, which points out the model’s effectiveness in isolating brain structures. Its performance is further validated in Fig 4 in terms of the original MRI images and their corresponding brain regions segments. These outcomes provide a solid ground for following segmentation tasks, e.g., gray matter extraction.

**Table 4:** Performance metrics of the standard U-Net (Vanilla U-Net) for the brain segmentation.

Model	Dataset	Dice	Precision	Recall	F1score
Vanila U-Net	ADNI	99%	99%	99%	99%

#### Phase 2: Gray matter segmentation.

**Fig 5:**
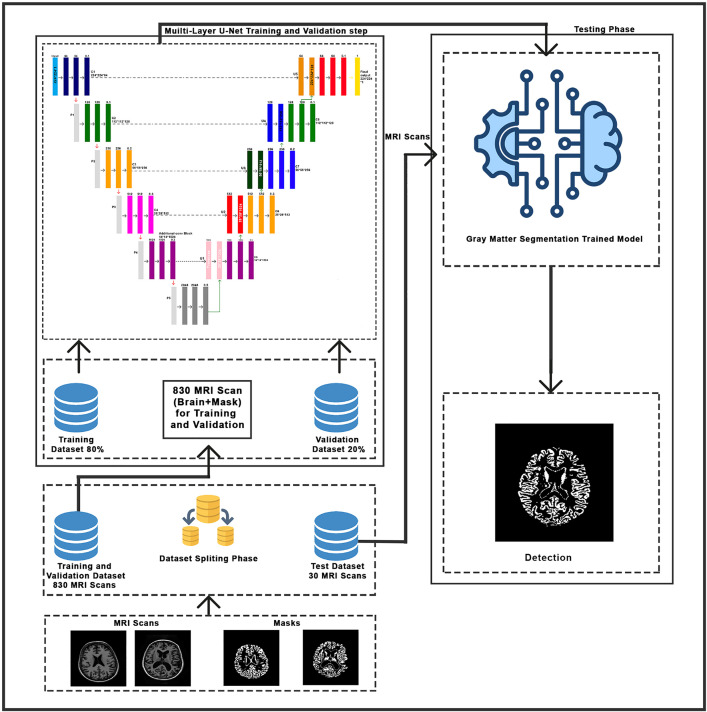
Proposed Multi-Layer U-Net model for gray matter segmentation.

**Fig 6:**
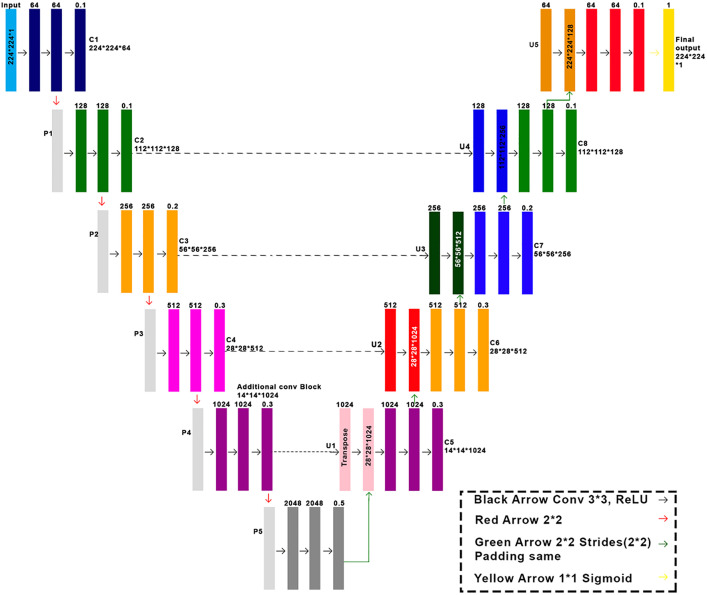
Architecture of Multi-Layer U-Net Model.

The second phase involves gray matter segmentation, where we choose the Multi-Layer U-Net to segment the gray matter in brain MRI due to its proven efficiency in medical image segmentation, especially in maintaining fine features. The greater depth of the multi-layer design of the Multi-Layer U-Net would enable better extraction and localization, both of which are important for capturing minor anatomical changes that underlie Alzheimer’s disease. The Multi-Layer U-Net achieves better segmentation accuracy than the Vanilla U-Net, primarily due to its increased depth and enhanced feature reuse. The Multi-Layer U-Net architecture was selected for gray matter segmentation due to its ability to better preserve structural detail in high-resolution brain MRIs. While the Vanilla U-Net was sufficient for broad brain segmentation, its limited depth hindered the precise delineation of gray matter regions. In contrast, the Multi-Layer U-Net, with its deeper architecture and increased receptive field, significantly enhanced segmentation accuracy. Although alternatives such as UNet++ and Attention U-Net offer improved context modeling, they were found to be more computationally intensive with negligible performance gains on our dataset. Therefore, the Multi-Layer U-Net offered the best trade-off between accuracy and efficiency for gray matter segmentation in our framework. Despite models such as SegNet being computationally desirable, they tend to fail at achieving comparable boundaries. Attention U-Net adds attention gates that would make the network pay more attention to important features, as a result, but in comparison tests (as part of the Multi-Layer U-NET implementation), it was outperformed by Multi-Layer U-Net and had greater computational overhead and lower segmentation accuracy as a result. Therefore, the Multi-Layer U-Net presents an acceptable balance between the effectiveness of segmentation and training speed and applies to the analysis of neurodegenerative disorders based on MRI. The outcomes of the quantitative segmentation are given in the Results section, where it is indicated that the Multi-Layer U-Net elevates region-specific accuracy, which is a parameter of critical importance in leading to the improvement of downstream classification performance. In this model, the output is the gray matter mask, and the input is the original MRI image. This approach was selected as it can segment fine-grained gray matter structures that are necessary for understanding. To fulfill the need for accurate segmentation, we trained and validated our model on MRI images using a Multi-Layer U-Net architecture, which is well-suited for capturing structural details in brain regions associated with Alzheimer’s disease overall structure of the model tackles both low-level and high-level spatial information, thus making the final model suitable to accurately separate gray matter structures, important biomarkers for the detection of Alzheimer related deviations. The model is trained and tested on 20% images of the ADNI Dataset to see how well it can predict gray matter segmentation masks. These predicted masks are an important input to subsequent classification tasks, allowing for the detection of small structural changes that are associated with Alzheimer’s disease. The model can precisely segment gray matter, which is important for the analysis of early diagnosis, focusing on a more targeted and refined analysis of neurodegeneration. The Proposed Multi-Layer U-Net model for gray matter segmentation is illustrated in Fig 5, and the architecture of the Multi-Layer U-Net Model is shown in Fig 6.

As the hierarchical features extracted by the encoder are reconstructed by the decoder in its parts, the skip connections fashion these parts to preserve fine-grained spatial details and enhance segmentation accuracy. We consider the multi-layer U-Net architecture containing these major components: the encoder, the decoder, the bottleneck, and the skip connection that performs different mathematical operations. The input image is progressively downscaled by convolutional blocks and max pooling layers, and additional features are progressively extracted at the hierarchical level. The convolutional operation, which occurs within the encoder, can be mathematically written down as:


ci=ReLU(Conv2D(Conv2D(pi−1,fi),fi)) (1)

Where ci signifies the output of the ith convolutional block, pi-1 denotes the output of the previous max pooling layer, and fi represents the number of filters in the ith block. The max pooling operation is expressed as:


pi=MaxPool2D(ci) (2)

Where pi is the output of the i-th max pooling layer. At the bottleneck stage, a convolutional layer

It is applied to capture intricate spatial details at the deepest level of the network. The decoder reconstructs the segmentation map by up-sampling the feature maps using transposed convolutions and concatenating them with the corresponding encoder feature to recover spatial details. This process is expressed as:


Ui=Concat(Conv2Dtranspose(ci−1,fi),cj) (3)

Where ui represents the output of the ith up-sampling layer, ci-1 is the output of the previous convolutional layer, cj corresponds to the feature map from the encoder, and fi denotes the number of filters. The final segmentation map is generated using a 1*1 convolutional layer, followed by a sigmoid activation function.


outputs=Sigmoid(Conv2D(cn,1)) (4)

Where cn is the output of the last convolutional layer in the decoder. For the optimization of model performance, the Adam optimizer is used to adjust the model’s weights (β) according to gradient updates. Moreover, a balancing cross-entropy loss function employing weighting factors for the contribution of positive and negative samples is also utilized to address the problem of class imbalance. It is defined by a loss function as follows:


Loss=−β·ytrue·log(ypred)−(1−β)·(1−ytrue)·log(1−ypred) (5)

Where Y_true_ denotes the ground truth mask, Y_pred_ represents the predicted mask, and *(β)* is a weighting factor that balances positive and negative sample contributions. While learning a segmentation network, the training images tend to include much more background than brain tissue, which causes the model to have a natural tendency to predict the background type default outcome. To avoid this, we apply class-balanced weights that increase the effect of the brain-tissue label over the background label. Such adaptation enables the model to detect anatomical features even of small sizes and outline the outlines of the gray matter with the highest accuracy. It is necessary to assess Alzheimer’s disease.

#### Phase 3: Classification.

**Fig 7:**
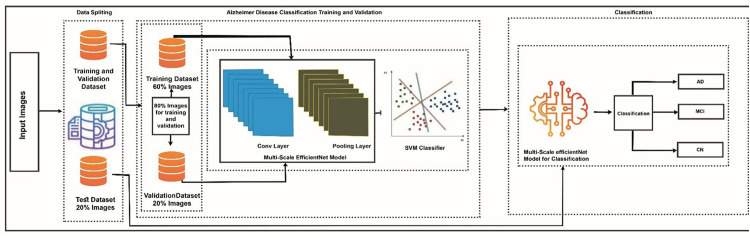
Proposed Alzheimer’s disease classification pipeline using Multi-Scale EfficientNet with SVM.

Compared to other widely used architectures like ResNet and DenseNet, EfficientNet offers superior efficiency with fewer parameters and FLOPs while maintaining higher accuracy [52]. ResNet benefits from residual connections and deep representation learning but tends to increase computational cost and may overfit with small datasets [53]. DenseNet encourages feature reuse and gradient flow, but its densely connected layers often introduce memory overhead and slow training [54]. EfficientNet, particularly the B0 variant used in our work, is lightweight and optimized through compound scaling. Making it ideal for medical imaging tasks that require fine-grained features and efficient computation. Recent studies have also shown that combining deep learning- based feature extractors with traditional machine learning classifiers can enhance performance, especially in medical imaging tasks where interpretability and precision are crucial [55]. Multi-Scale EfficientNet offers a scalable and efficient way to extract multi-level structural features from MRI scans. However, instead of relying solely on its end-to-end classification layers, we opted to pair it with a Support Vector Machine (SVM) classifier. This hybrid approach leverages the feature extraction power of Multi-Scale EfficientNet and the strong decision boundaries of SVM, which is particularly effective in complementing data augmentation strategies and enhancing classification robustness in a limited medical imaging dataset [56]. Comparative experiments demonstrated that this fusion model outperformed a standalone deep learning classifier in terms of accuracy and generalization, validating the robustness of the chosen architecture. Therefore, it is time to distinguish between the two major phases of the semi-supervised model we are discussing. The last element of the analysis is the classification stage. In this case, a hybrid solution has been employed that combines the effective convolutional-based backbone, namely Multi-Scale EfficientNet, with a classical approach based on the kernel, specifically the Support Vector Machine (SVM). The feature maps provided by EfficientNet are directly passed on to the SVM, which in turn maps the vectors to one of three mutually exclusive categories: Alzheimer’s Disease (AD), Mild Cognitive Impairment (MCI), and Cognitively Normal (CN). The combination of the potential of EfficientNets to scale with the demonstrated ability of SVM to define a robust decision boundary emphasizes the accuracy of the whole classification process. The proposed Alzheimer’s disease classification pipeline using Multi-Scale EfficientNet with SVM is illustrated in Fig 7.

**Fig 8:**
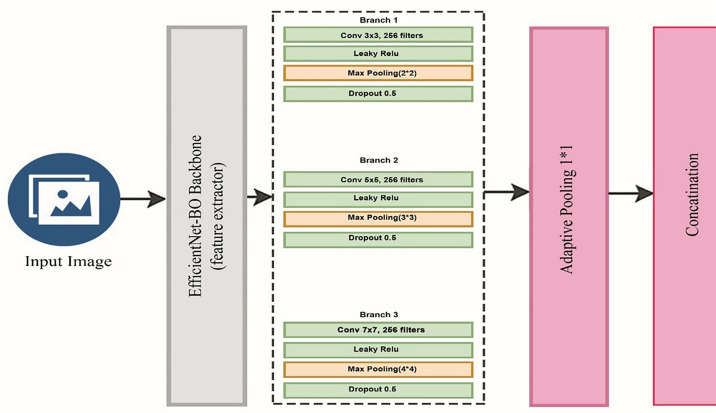
Architecture of Multi-EfficientNet.

The feature extractor was made up of the multi-scale version of the EfficientNet-B0 architecture. Three parallel convolutional branches with multiple scales of receptive fields of different kernel sizes of 3 x 3, 5 x 5, and 7 x 7 were used to replace the final fully connected layer. All the branches contained a max pooling, a LeakyReLU activation layer, and a dropout to regularize. The proposed architecture of Multi-scale EfficientNet is illustrated in Fig.8.

computingℝ¹²⁸⁰ˣ^H^ˣ^W^ denotes the feature map from the EfficientNet backbone, then the three branches compute:


$$F_{1}=Pool\left(\sigma \left(W_{1}*F+b_{1}\right)\right),$$ (6)

$$F_{2}=Pool\left(\sigma \left(W_{2}*F+b_{2}\right)\right),$$ (7)

$$F_{3}=Pool\left(\sigma \left(W_{3}*F+b_{3}\right)\right),$$ (8)

In which * represents convolution, sigma (σ) represents LeakyReLU activation, and Pool represents max pooling, which is then followed by adaptive average pooling. The outputs that were produced were concatenated and flattened:


$$F\_concat=Flatten(Concat\left(F_{1},F_{2},F_{3}\right))$$ (9)

The feature vectors thus obtained were then used to train an SVM classifier using an RBF kernel. The definition of decision function was as follows:


$$\begin{array}{c} f(x)=sign(\sum_{i}\alpha_{i}y_{i}exp\left(-\gamma |\left|x_{i}-x\right|{|}^{2}\right)+b) \end{array}$$ (10)

In which x is the set of extracted features and xᵢ are support vectors, yᵢ ∈ {+1, 1}, and γ is the kernel coefficient. The standard scaler was used before the vectors were fed into the SVM to make the classification model robust. In this study, feature extraction is performed from the final convolutional layer of Efficient Net B0 using its extract features method. This stage captures high-level semantic representations of the brain MRI, which are highly informative for differentiating between AD, MCI, and CN. By processing these deep features through three parallel convolution branches with different kernel sizes (3 x 3, 5 x 5, and 7x7), the network is also able to capture both fine-grained local structures and broader contextual patterns. Training of the model was done using cross-entropy loss as the optimization objective:


$$L\_CE=-\sum_{i}y_{i}\text{log}({ŷ}_{i})$$ (11)

Where yᵢ is the true label and ŷᵢ is the class probability as computed via softmax. It was trained with a learning rate of 0.0001. Optimal hyperparameters were obtained using Grid search, which produced the highest measure of performance with kernel = ‘rbf, C=10, and gamma=’scale’. Cross-validation was used to evaluate the model, and several measures referencing robustness and generalization were observed, including accuracy, precision, recall, and F1-score. The Grid Search function used accuracy as the primary scoring metric for evaluating each parameter combination. Table 5 shows SVM Hyperparameter Tuning results using Grid Search.

**Table 5:** SVM Hyperparameter Tuning Results Using Grid Search.

Kernel	C	Gamma	Accuracy (%)
RBF	10.0	Scale	97.43%
RBF	10.0	Auto	97.43%
Liner	0.1	Scale	96.53%
Linear	0.1	scale	96.53%
Linear	10.0	Auto	96.53%
RBF	1.0	scale	96.22%
RBF	0.1	Auto	91.15%

### Experimental setup

All experiments were conducted using Google Colab, utilizing a cloud-based NVIDIA Tesla T4 GPU (16 GB VRAM). The segmentation model was implemented in TensorFlow 2.12 and Keras 2.12, while the classification model was developed using PyTorch. Key supporting libraries included NumPy, OpenCV, Matplotlib, scikit-learn, and TensorFlow’s ImageDataGenerator for data augmentation. For segmentation, the model was trained with a batch size of 8, 100 epochs, and a learning rate of 1e-4 using the Adam optimizer. All the experiments were executed with a fixed random seed (numpy.random.seed(42), tensorflow.random.set_seed(42)) for deterministic behaviour across runs. For classification, we used a batch size of 32 and the Adam optimizer with default PyTorch settings. Hyperparameter tuning for SVM was performed using GridSearchCV, and fixed random seeds were used to ensure reproducibility. Detailed hyperparameter settings for both segmentation and classification models are provided in Table 6:

**Table 6:** Details of Hyperparameters.

Component	Parameter	Value/ Range
**Segmentation**	Model	Multi-Layer U-Net
	Optimizer	Adam
	Learning Rate	1e-4
	Batch Size	8
	Epochs	100
	Loss Function	Balanced Cross-Entropy
**Classification**	Model	Multi-Scale EfficientNet-B0 + SVM
	Optimizer	Adam
	Learning Rate	1e-4 (EfficientNet)
	Batch Size	32
	SVM Kernel	RBF, Linear (tuned via GridSearchCV)
	SVM C	[0.1, 1, 10]
	SVM Gamma	[‘scale’, ‘auto’]

When we discuss the performance of the presented model of deep learning classification of Alzheimer’s disease, we use well-founded indicators to assure our results reflect the strength of the given model in the ability to correctly discriminate between classes of the disease.

### Accuracy

Accuracy refers to the total %age of accuracy of the model. It is a ratio between correctly predicted cases to the overall number of predictions.


Accuracy=TP+TN/TP+TN+FP+FN

### Precession

Precision is the accuracy of positive outcomes. It indicates the %age of true positives concerning the total positive cases.


Precision=TP/TP+FP

### Recall (Sensitivity)

Recall is the power of the model to locate all the applicable cases in a data set. It is an indication of the number of real positive cases that were rightly predicted.


Recall=TP/TP+FN

### F1-Score

F1-score is a harmonic mean of the precision and the recall; it is a single indicator of the two items.


F1−Score=2×Precision×Recal/Precision+Recall

### Dice Similarity Coefficient (DSC) and Intersection over Union (IoU)

Both are popular segmentation performance measure indices. Dice coefficient is a measure of overlap of the predicted segmentation to the actual one, P and Ground truth G, denoting.


Dice=∣P∣+∣G∣2∣P∩G∣

Where ∣ *P* ∩ *G*∣, the intersection of prediction and ground truth, is 1/8 of pixels: 1/8(png). Dice takes the values between 0 (no overlap) and 1 (perfect overlap). It measures the size of the intersection over the union, known in the literature as the Jaccard Index or IoU.


IoU=∣P∪G∣∣P∩G∣

Both measures have different complementary views on segmentation quality, where Dice is well suited to measure the proportion of overlapping in similar proportions, and IoU is well suited to intersect in proportion to the sum of the areas.

### Workflow summary

In the computational pipeline that we propose, two major steps are noted. In the first step, we pre-processed the original 3D MRI volumes using SPM12 to create 2-dimensional axial slices with the representation of 2D gray matter. These axial slices were then divided into a Multi-Layer U-Net-based architecture to perform the segmentation. In stage two, the segmented and preprocessed gray matter images were piped to our Multi-Scale EfficientNet model, which was used to extract features. A Support Vector Machine (SVM) was used then to classify the resulting feature representation and evaluate the performance through accuracy, precision, recall, as well as F1-score. The Dice coefficient and a balanced cross-entropy loss were also used as a measure of segmentation performance.

### Phase 4: Explainable AI using saliency map

**Fig 9:**
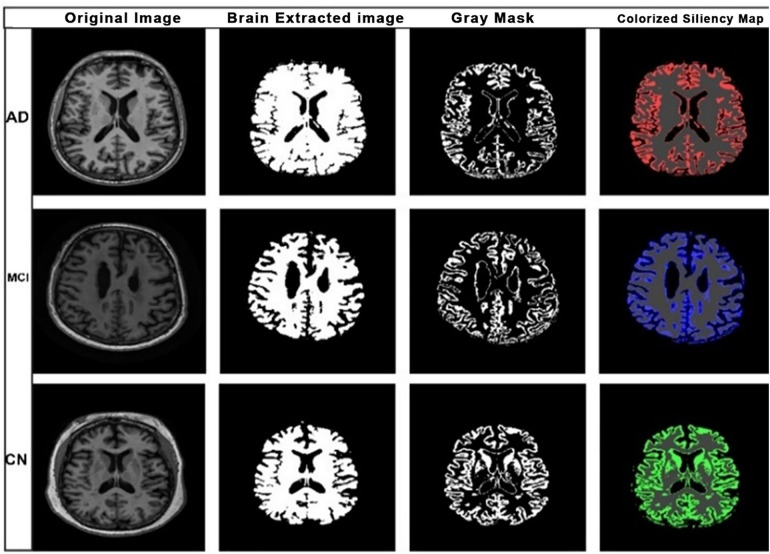
XAI Saliency map visualization for the proposed model.

To increase transparency and trustworthiness, a saliency map is incorporated into our Alzheimer’s classification model, based on Explainable AI (XAI) techniques, to visualize the brain regions most important for disease classification. XAI is significant for medical imaging because it enables us to go beyond having accurate predictions to explain why the model made its decisions. It is necessary to understand this for building trust in the models’ predictions and for use by clinicians to gain actionable insights. In our implementation, the generation of the saliency map was run following the successful training and testing of the models. In such a way, the accuracy, precision, recall, as well as F1-score values did not change when the saliency maps were created or not. The motive behind the creation of the saliency maps is solely interpretation, and only to form an idea of the qualitative examination of the following stage of clinical authentication. As illustrated in Fig 9

The original MRI scan, the segmented brain, the isolated gray matter, and the final colorized saliency map. This colorization scheme provides an intuitive color reflectance to disease severity (where red = AD, blue = MCI, and green = CN). In the colored version of the images, higher saliency values correspond to brighter colors, indicating a stronger influence on the classification. It also employs the use of Gaussian-blurred saliency maps with edge detection applied to sharp contrast anatomical structures in gray matter. Our choice to use saliency maps is deliberate, as Alzheimer’s disease-related degeneration often manifests as subtle, localized patterns best captured at the pixel level. This pixel-focused interpretability allows the model to highlight disease-relevant signals in gray matter with high spatial resolution, which is particularly suitable for brain MRI data. While saliency maps offer pixel-level visualization. However, our saliency-based approach has proven sufficient in this study by revealing meaningful spatial patterns across disease stages, and additional techniques were not required to meet our interpretability objectives. We conduct quantitative analysis on saliency maps to further analyze these visual insights. The importance of this step is that we can objectively measure and compare the model’s focus on different brain regions across different disease stages. We then created four regions (by dividing each image) and calculated the saliency average value for AD, MCI, and CN cases, respectively. These results are presented in Table 7. This quantitative analysis shows how the model pays attention to different regions and disease stages. For example, region 3 has higher saliency values for CN than AD, implying that this region is important in distinguishing CN from AD. Likewise, the variations in saliency values across MCI and CN suggest unique spatial activation patterns.

**Table 7:** Average Saliency Map Results.

Class	Region 1	Region 2	Region 3	Region 4
AD	0.0859	0.0861	0.1163	0.1159
MCI	0.0961	0.0946	0.1032	0.1112
CN	0.1124	0.1093	0.1255	0.1272

This region-wise saliency analysis provides a stage-wise interpretation of how the model’s focus changes between CN, MCI, and AD. While not based on temporal (longitudinal) MRI, this static visualization offers insight into the spatial progression of Alzheimer’s classification.

The detailed analysis reveals not only the model’s decision-making process but also its ability to identify clinically relevant biomarkers. We pursue the gap between deep learning diagnostics and clinical applicability by visualizing the model’s focus on clinically related brain regions and using quantitative measures of regional activation in the decision-making process. Such an approach meets the principles of saliency visualization, whose goal is to identify which features contribute the most to the model’s prediction. Wang et al. [24] exemplified this approach in studies employing similar techniques to interpret deep learning models in medical imaging. These XAI techniques allow for better comprehension of model performance and how it may detect biomarkers associated with AD and MCI progression. Since our classification model uses a multi-scale EfficientNet architecture, it inherently captures features at different spatial resolutions. This allows our interpretability analysis, particularly the saliency-based regional activation quantification, to reflect multi-scale patterns that align with anatomical variability across AD, MCI, and CN. In the present study, the gray matter structures were clearly emphasized in the saliency maps, which is in agreement with the previous literature about gray matter volume reduction as the major anatomic pathology of Alzheimer’s disease (AD). This kind of pattern strengthens the biological salience of the predictive results of the model. More so, the distinction between AD and mild cognitive impairment (MCI) was less discriminating in comparison with AD and cognitively normal (CN) controls, reflecting the linear medical spectrum that exists between MCI and early AD.

## Results

The decisions in this study are based on Magnetic Resonance Imaging (MRI) scans in the Digital Imaging and Communications in Medicine (DICOM) format, where both the imaging data and essential metadata are stored within the DICOM header. Decisions of this study make use of Magnetic Resonance Imaging (MRI) scans in the Digital Imaging and Communications in Medicine (DICOM) format, in which the data and mandatory metadata are contained within the DICOM header. The metadata in this includes especially valuable information, such as patient identification, scanning trajectory, slice thickness, spatial resolution, as well as what type of imaging modality was used. The instance numbers of the scan parts are also contained in the DICOM header, sorting the multiple slices systematically. The ability to reconstruct 3D brain structures with high accuracy is vital for assessing neurodegenerative patterns with Alzheimer’s disease, and errors in this indexing will affect the accuracy of this process. In this research, the dataset used is collected from the Alzheimer’s Disease Neuroimaging Initiative (ADNI) with three diagnostic classes: Alzheimer’s disease (AD), Mild Cognitive Impairment (MCI), and Cognitive Normal (CN). It begins with a multi-layer U-Net model used to segment gray matter to obtain fine-grained structural features that are useful to detect Alzheimer related abnormalities. As early indicators of Alzheimer’s progression, gray matter density and structure alterations are found via this segmentation phase. Dice loss is used for training the model, and the Adam optimizer is used to optimize it, resulting in a robust performance in segmentation. Dice score, Intersection over Union (IoU), Sensitivity, and Precision all serve to validate the model’s ability to properly delineate gray matter regions. After segmentation, the classified gray matter images are used as input for a hybrid deep learning pipeline that **uses Multi-Scale** Efficient Net **for Feature extraction** and **SVM for classification**.

### Result of gray matter segmentation phase

A multi-layer U-Net is proposed, which effectively segments gray matter regions from MRI scans more accurately than existing approaches. The two-step algorithm used for segmentation begins by training and validating models on the ADNI dataset, followed by the prediction of gray matter masks for unseen MRI scans. This model uses three encoder and decoder layers with multiple skip connections to improve feature retention and preserve spatial information. All the reported metrics were calculated on the test set. Table 8 presents a comparison of these segmentation models and shows the superiority of the proposed method. The performance of Vania U-Net, Attention U-Net, has a Dice Score of 89.85% ± 0.10. Other methods, such as cGAN, have a dice score of 78% Multitask Deep CNN has a Dice Score of 87% and DIDL also has a Dice score of 87% which are outperformed by our proposed method, Multi-layer U-Net, which has a Dice score of 90.06% ± 1.76%. This is significantly higher than Vanilla U-Net and Attention U-Net, both have a Dice score of 90%.

**Table 8:** Comparative evaluation of gray matter segmentation using Multi-Layer U-Net, Vanilla U-Net, Attention U-Net, and state-of-the-art methods. The results of the proposed model are calculated on the test set.

Model	Dataset	Loss function	Accuracy	Dice	Recall	Precision	F1-Score
U-Segnet [35]	IBSR	–	–	89%	80%	82%	83%
cGAN [34]	T2-Flair, T1 Images	–	–	76%	84%	69%	–
Multi-Task Deep CNN [25]	ADNI	–	–	87%	–	–	–
Attention U-Net	ADNI	Balanced Cross-Entropy	97%	90%	89%%	87%	90%
Vanila U-Net	ADNI	Balanced Cross-Entropy	97.35% ± 0.25	89.85% ± 0.10	87.28% ± 0.51	91.90% ± 1.34	89.85% ± 0.10
Proposed Multi-Layer U-Net	ADNI	Dice – Loss	88.64% ± 8.35	71.24% ± 16.42	91.98% ± 4.08	62.81% ± 24.42	71.24% ± 16.42
Proposed Multi-layer U-Net	ADNI	Balanced Cross-Entropy	97.66% ± 0.31%,	90.06% ± 1.76%,	87.25% ± 3.56%,	92.74% ± 3.71%,	90.06% ± 1.76%,

Moreover, to provide a measure of variability and robustness, we run the model five times and report the average metrics along with their standard deviations. The model achieved an accuracy of 97.66% ± 0.31%, Dice coefficient of 90.06% ± 1.76%, a recall of 87.25% ± 3.56%, a precision of 92.74% ± 3.71%, and F1-score of 90.06% ± 1.76%, which is an improvement in delineation of the gray matter structures for the Alzheimer’s disease diagnosis. In comparison, we have also performed our experiments with the change of loss function, where we changed balanced cross-entropy to dice loss, but we did not obtain better results. Fig 10 and Fig 11 show the qualitative segmentation results produced by the proposed Multi-Layer U-Net and the Attention U-Net Model, respectively. The visual comparison highlights the improved gray matter boundary delineation achieved by the proposed better segmentation performance permits more accurate structural analysis of MRI scans in the service of downstream classification of Alzheimer’s disease detection.

**Fig 10:**
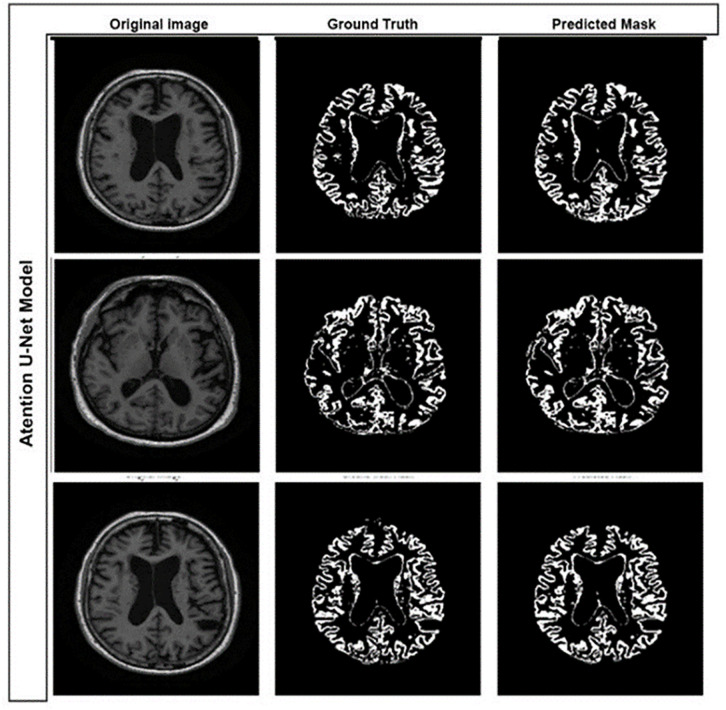
Gray matter segmentation results using the Attention U-Net model.

**Fig 11:**
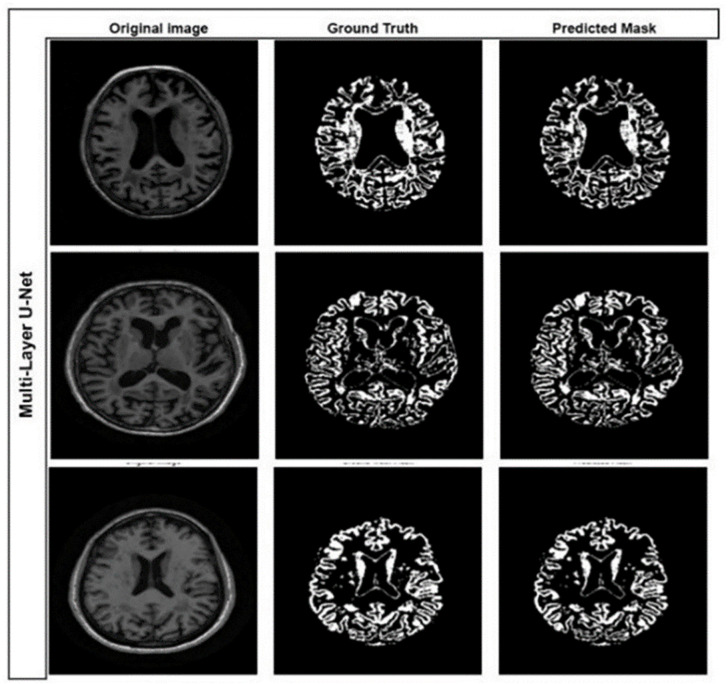
Gray Matter segmentation results using the proposed Multi-layer U-Net Model.

## Result of classification phase

**Fig 12:**
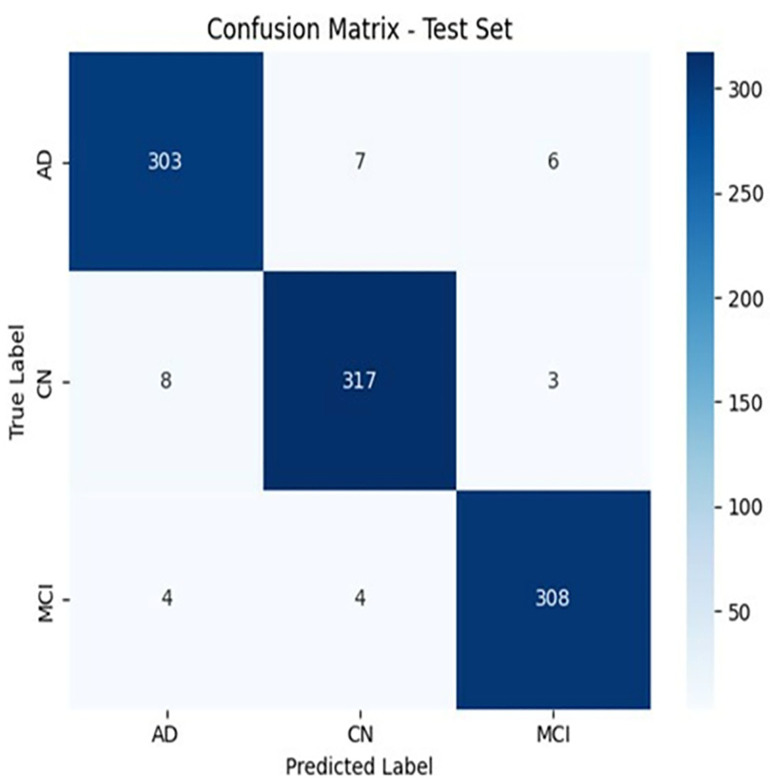
Confusion Metrics: Presenting classification results on the test set.

To ensure statistical roundness, all the experiments were repeated five times, and the reported metrics represent the mean ± standard deviation across these runs. We evaluated the generalization performance of the Multi-Scaled Efficient Net with an SVM model to determine how well it could distinguish the data from the test dataset. It contains AD, CN, and MCI classes. The classification report generated on the test set showed a high classification accuracy of 96.44% ± 0.91%, suggesting high classification accuracy by the model. The high accuracy is also well supported by the metrics, which are as high as 96.37% ± 0.016%, 96.06% ± 0.014%, and 96.61% ± 0.014% precision; 96.98% ± 0.018%, 95.75% ± 0.017%, and 96.72% ± 0.013% recall; and 96.68% ± 0.011%, 95.84% ± 0.014%, and 96.63% ± 0.011% F1 scores for every class. More specifically, AD classification achieved a precision of 96.37% ± 0.016%, a recall of 96.98% ± 0.018% and an F1 score of 96.68% ± 0.011%, which means that it is very good at correctly identifying AD cases without many false positives. The performance of the CN class was good and exhibited a precision of 96.06% ± 0.014%, a recall of 95.75% ± 0.017%, and an F1 score of 95.84% ± 0.014%. This denotes reasonable performance in terms of identifying CN individuals. On the MCI class, the model achieved a precision of 96.61% ± 0.014%, a recall of 96.72% ± 0.013%, and an F1-score of 96.63% ± 0.011%, which accurately describes the precision in identifying MCI, with a slight drop in recall. In further demonstrating the per-class performance on the test set, Table 9 shows precision, recall, and F1-score by the classes. Analysis of the results shows the model behaves consistently between AD, CN, and MCI classes, but with an extremely high recall on the CN class and roughly equal performance on AD and MCI. The confusion matrix in Fig 12 gives an intuitive visual representation of the classification results, with the correct results presented on the diagonal and the misclassifications of the diagonal. According to the findings, most of the samples of each category were correctly identified, with a few incorrect identifications being recorded, such as 7 CN samples being identified as AD and 6 MCI samples being identified as AD. This is because minimal cross-class confusion shows that the model successfully classifies the different conditions of AD, MCI, and CN subjects very well, even when the inter-class similarity may lead to overlap.

**Table 9:** Class-wise performance on the test set.

Class	Precision	Recall	F1-Score
AD	97.18% ± 1.14%	97.90% ± 0.77%	97.74% ± 0.63%
CN	97.78% ± 0.29%	97.49% ± 1.34%	97.78% ± 0.79%
MCI	97.03% ± 1.10%	97.25% ± 0.99%	97.54% ± 0.69%

These findings identify that in even the more complex MCI group, which sometimes overlaps with both the AD and CN features, the model is very precise and high recall, which shows great strength of performance in differentiating between the diseases. Following this, a comparative analysis was conducted to evaluate the performance of the model against the existing state-of-the-art approaches.

Table 10 presents the comparison of the test set results with state-of-the-art models. Alzheimer’s disease classification has been evaluated using various deep learning and machine learning models. Strong classification capabilities were performed by the different Explainable CNN + MKSCDDL and the Hybrid Model. Although such traditional machine learning models as Multi-Layer Perceptron and Logistic Regression looked good, but lacked the tenacity of deep learning-based models. It was found that DenseNet201 is lacking in feature extraction from MRI scans, as indicated by the lower classification accuracy. While Gradient Boosting and ResNet18 both provided moderate performance, ResNet18 was particularly struggling with separating the stages of Alzheimer’s disease. Significant improvement was obtained by the proposed Multi-Scale Efficient Net, demonstrating its effectiveness in capturing complex patterns and improving reliability classification.

**Table 10:** Comparison of the Classification performance of the proposed model calculated on the test set with state-of-the-art Approaches.

Model	Dataset	Year	Accuracy	Recall AD MCI CN	Precision AD MCI CN	F1-Score AD MCI CN
ResNet18 [6]	OASIS	2025	74%	- - -	- - -	- - - -
ViT [56]	ADNI	2022	78%	60% - -	83% - -	70% - -
Explainable CNN+MHSCDDL [55]	ADNI	2024	–	98% 94% 88%	96% 91% 89%	97% 92% 86%
Hybrid Model	ADNI	2024	94%	− 88% 89%	− 80% 93%	− 84% 91%
Logistic Regression	–	2024	81%	- - -	- - -	- - -
Multi Layer Preceptron	–	2024	76%	- - -	- - -	- - - -
Gredient Bossting	–	2024	83%	- - -	- - -	- - - -
DenseNet201	ADNI	–	53%	23% 39% 99%	44% 32% 78%	32% 35% 39%
Proposed Multi-Scale EfficientNet + SVM	ADNI	–	96.44% ± 0.91%	96.98% ± 0.018%, 95.75% ± 0.017%, 96.72% ± 0.013%	96.37% ± 0.016%, 96.06% ± 0.014%, 96.61% ± 0.014%	96.68% ± 0.011%, 95.84% ± 0.014, 96.63% ± 0.011%

Conducting a careful analysis of the additional experiments shown in Table 11, one can see that the extra experimentation proves the importance of design decisions upon the accuracy of classification once more. The findings directly show that the combination of multi-scale feature extraction with gray matter segmentation yields the best figure (97.78% + /- 0.54%) compared to its one-scale version and the known benchmark, provided by the model such as MobileNetV2. Given that the decrease in the measure of precision without data augmentation is a significant result of 97.78% ± 0.54% compared to 89.48% ± 1.49%, the role played by augmentation in the data generalization of the model can hardly be ignored. Similarly, the Accuracy is lower when trained on whole-brain volumes intact, as opposed to the selection of gray matter. Thus, the segmentation of the affected areas is justified. Together, all these findings emphasize the fact that the quality of the segmentation, the magnitude of the feature extraction, and the strategy to augment them are critical factors for classification.

**Table 11:** Comparative performance analysis of different architectures.

Method	Gray Matter images	Whole brain/Raw MRI images	Accuracy	Recall	F1-Score	Precession
Single Scale	Yes	No	95.42% ± 0.73%	95.35% ± 0.74%	95.36% ± 0.74%	95.40% ± 0.74%
Proposed Multi-scale Efficient Net	Yes	No	97.78% ± 0.54%	97.78% ± 0.54%	97.77% ± 0.54%	97.76% ± 0.54%
No-Augmentation	Yes	No	89.48% ± 1.49%	89.57% ± 2.58%	89.53% ± 2.03%	89.74% ± 2.50%
Mobile NetV2 (baseline)	Yes	No	95.14% ± 1.56%	95.90% ± 1.89%	95.92% ± 1.50%	95.99% ± 1.69%
Proposed Multi-scale Efficient Net	No	Yes	96.35% ± 1.30	95.91% ± 1.23	95.88% ± 1.27	95.88% ± 1.28

### Statistical significance testing.

To assess the statistical significance of the observed performance improvement, the McNemar test was applied to compare the classification results of the proposed hybrid model against the Efficient Net B0 baseline model. The test yielded a McNemar statistic of 374.21 and a p-value of 2.26 × 10^⁸³, which is far below the conventional threshold of 0.05, indicating a statistically significant difference in model performance. The result supports the conclusion that the hybrid model offers meaningful and non-random improvement in classification accuracy for Alzheimer’s disease classification.

### ROC curve

**Fig 13:**
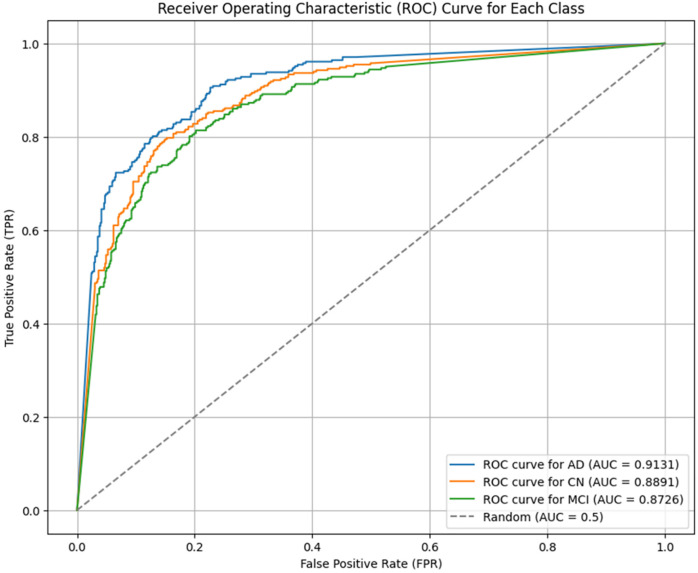
ROC curve for the proposed Classification model across AD, MCI, and CN.

Furthermore, the Receiver Operating Characteristic (ROC) curve for AD, MCI, and CN was created to judge the performance of classification models. ROC curves were made for each class, Alzheimer’s Disease (AD), Cognitively Normal (CN), and Mild Cognitive Impairment (MCI), as a function of the decision threshold by calculating False Positive Rate (FPR) vs True Positive Rate (TPR). To get just one scalar value as a summarization of the model’s ability to discriminate for each class, the Area Under the Curve (AUC) was computed. The ROC-AUC metric was calculated on the test set in a one vs rest manner for each class. Fig 13 shows that the model can achieve high AUC values in all categories, which are 0.9131, 0.8891, and 0.8726 for AD, CN, and MCI, respectively, meaning its classes are highly separable. The ROC curves also demonstrate that the classifier has a high sensitivity and specificity for different operating points and, therefore, is robust to the disease stages. Additionally, optimal decision thresholds for each class were found by maximizing the difference between TPR and FPR and making the model clinically applicable. Thus, ROC curves offer complete visualization and a quantitative measure of a model’s performance, which is essential for validating its use in real-world clinical diagnostics.

### Ablation study

To assess the role of segmentation and the choice of the classifier, we conducted a comparative study of 3 arrangements: (i) U-Net segmentation and Multi-Scale EfficientNet classification by SVM, (ii) Multi-Scale EfficientNet classification without the segmentation step and SVM, and (iii) end-to-end Multi-Scale EfficientNet classification. Each of the experiments was conducted using the same data partitions and augmentation strategies, and each runtime was repeated five times. The results have been outlined in Table 12. The setup with Multi-Scale EfficientNet classification with SVM and without a segmentation stage condition had the ultimate average accuracy rate (95.84% ± 1.30). However, the complete pipeline U-Net segmentation, Multi-Scale EfficientNet classification, and SVM has an accuracy rate of (97% ± 0.54%), and it gave anatomically centered features, which best fit clinical workflow approaches, interpretability, and reduce reliance on non-pathology-specific image components. However, removing the U-Net segmentation and SVM post-processing, the end-to-end Multi-Scale EfficientNet network produced an accuracy that had dropped significantly (67.94% ± 9.89%). These findings reiterate the mandatory presence of the SVM in the use of extracted features and defining solid decision boundaries.

**Table 12:** Ablation Study Result.

Model	Accuracy
Full pipeline	97.78% ± 0.54%
Multi-Scale Efficient Net + SVM (No Segmentation)	95.84% ± 1.30
End to End Muilti- Scale Efficient Net (No Segmentation)	67.94 ± 9.89%

## Conclusion

I would like to describe the hybrid model offered in this paper. A multi-layer U-Net model was trained to distinguish gray matter, and the cross-section was passed to an encoder-decoder system built with EfficientNet, followed by a Support Vector Machine to binarily categorize Alzheimer’s disease. The assessment in ADNI using the dataset resulted in a Dice coefficient of 91.52% in the segmentation aspect, outranking the Vanilla U-Net (90%) and Attention U-Net (90%), therefore proving the effectiveness of the model in writing off brain regions of interest to the disorder. Performance of the classification on the test set produced an accuracy of 96%, which is much higher than the accuracy of each of the architectures separately. The explanation of diagnostic decisions was also improved using an explanation mechanism (Explainable AI, XAI) with alterations in saliency maps, resulting in knowledge visualization of the brain regions behind the decision-making in each diagnosis. In sum, the findings suggest that the hybrid design provides a competent trade-off between the accuracy of diagnostic findings and model interpretability of MRI-based Alzheimer’s disease identification.

There are limitations of the present study. Its output still must be reproduced on autonomous cohorts like OASIS and AIBL. Also, longitudinal stage tracking and temporal development were not parts of this research.

### Future work

The upcoming study will intentionally spread the interpretative range of the current models. Among the milestones is validating transfer-learned architectures with a range of clinical datasets, including OASIS, as well as the AIBL cohort. We will use confidence intervals, multi-run averaging, and rigorous hypothesis testing to achieve statistical strength in the tests conducted. In addition, we will explore the latest XAI techniques--Grad-CAM, SHAP, and Integrated Gradients--in order to get a more detailed understanding of the model explanation. Finally, over time, the aim will be to generate longitudinal models that can be used to monitor the changing course of the degenerative condition on the prognostic continuum.
